# Prevalence of Cardiotoxicity Secondary to Trastuzumab in Patients with HER-2-Positive Breast Cancer in Southeast Mexico

**DOI:** 10.3390/reports7030076

**Published:** 2024-09-14

**Authors:** Luz I. Pascual-Mathey, Midory I. Velez-Figueroa, Joel J. Díaz-Vallejo, Gustavo Mendez-Hirata, Gustavo F. Mendez-Machado

**Affiliations:** 1 Laboratory of Molecular Biology, Faculty of Pharmaceutical Biological Chemistry, Veracruzana University, Jalapa 91190, Mexico; joediaz@uv.mx; 2 UMAE Specialty Hospital 14, National Medical Center Adolfo Ruiz Cortines, IMSS, Veracruz 91810, Mexico; izareth179@gmail.com; 3 School of Medicine, Panamericana University, Mexico City 03920, Mexico; 0208045@up.edu.mx; 4 Cardiology, General Zone Hospital 11, IMSS, Xalapa 91000, Mexico; gusmendez@uv.mx

**Keywords:** Her2-positive breast cancer, trastuzumab, cardiotoxicity, prevalence

## Abstract

In Mexico, breast cancer (BC) is the principal cause of death in women over 30 years old, with an annual mortality rate of 14.61 deaths per a 100,000 population. Chemotherapy, in combination with trastuzumab (TTZ), improves the survival of cancer patients; however, cardiotoxicity (CT) is the principal consequence. CT prevalence occurs between 10% and 30% of patients; however, there are no data about the prevalence of CT in the Mexican population. This study aims to establish the prevalence of CT in patients treated with anti-HER-2 therapy among BC women in southeast Mexico. A retrospective cross-sectional study was carried out from January 2015 to July 2019. The records of 46 patients diagnosed with HER-2-positive BC who attended the Mexican Social Security Institute in the Ambulatory Care Medicine Unit were analyzed. The diagnostic criterion for CT was a decrease in LVEF > 10% from baseline or a final LVEF < 53%. CT prevalence was observed in 19 (41.3%) of women with cancer, with an average decrease in LVEF of 13%. In the population, we found an association between weight, surface area, and the loading dose of TTZ with CT. Nutritional follow-up and the administration of cardioprotective drugs are necessary to recover LVEF and avoid cardiovascular failure in women with BC and survivors.

## 1. Introduction

With the use of a novel class of drugs for cancer implemented in recent decades, the number of surviving patients has increased. In the United States, the number of patients with a history of cancer was approximately 19 million in 2022, with an estimated substantial increase of one in three (30%) of all new female cancers every year [1,2]. 

In Mexico, breast cancer (BC) is the first cause of disease in women over 30 years of age, reporting an annual mortality rate of 14.61 deaths per a 100,000 population with an upward trend [3]. Currently, chemotherapy (QT), in combination with new antibody–drug conjugates (ADCs), improves the survival of cancer patients. However, adverse side effects on the cardiovascular system are observed, including heart dysfunction and cardiotoxicity (CT) [4,5]. Accordingly, anthracyclines and the anti-human epidermal growth factor receptor 2 (HER-2) monoclonal antibody represent a successful therapeutic strategy for BC; however, they are associated with CT [6,7,8]. 

Anthracyclines have cytostatic and cytotoxic actions with no reversible dose-dependent cardiac toxicity [5]; trastuzumab (TTZ) is a monoclonal antibody used in the treatment of HER-2-positive BC with no dose-dependent reversible toxicity [5]. The HER-2 family belongs to the human epidermal growth receptors involved in the pathogenesis of breast cancers where HER-2 is overexpressed. In this sense, 16 to 27% of women with BC overexpress HER-2 with significant prognostic implications. TTZ is a standard in the management of HER-2-positive BC patients. Its use improves the survival of patients, with a longer time until disease progression of 7.4 vs. 4.6 months and a higher objective response rate [9,10,11]. However, the prevalence of cardiotoxic effects associated with the use of these therapies has increased [4].

Cardiotoxicity is a cardiac muscle dysfunction associated with exposure to antineoplastic treatments, which can progress to heart failure. Concerning the above, CT secondary to chemotherapy and TTZ is reported in breast cancer patients, with the prevalence of symptomatic heart failure ranging from 4% to 17% in clinical trials. More interestingly, the incidence of cardiac failure increases with the concomitant administration of TTZ–anthracyclines or other cytostatic ones (27%) than when administered alone (7%) [4,5]. The diagnosis of CT includes the structural changes that condition the left systolic ventricular function by analyzing the left ventricle ejection fraction (LVEF) [10,11,12,13]. The diagnostic criterion used to define CT is considering a drop in LVEF > 10% from baseline or a final LVEF < 53% (ESMO) [7,14]. In Mexico, a recent study showed alarming data with a prevalence of cardiotoxicity, evaluating both echocardiography and troponin in 23 patients treated with a combination of TTZ and anthracyclines from a population of 50 women with breast cancer [15]. Therefore, this clinical study aimed to determine the prevalence of cardiotoxicity in breast cancer patients treated with anti-HER2 therapies in an ambulatory unit in the state of Veracruz, Mexico.

## 2. Results

**Figure 1:**
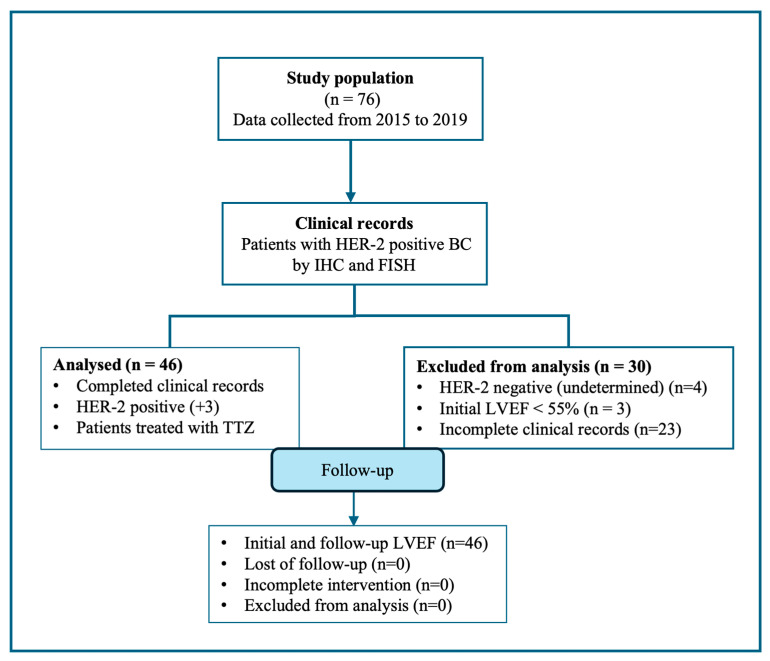
The flow diagram of the retrospective cross-sectional study performed is shown. After selection, thirty records were excluded; records of forty-six HER-positive BC patients were included in this study.

Seventy-six breast cancer patients with the overexpression of HER-2 receptors by immunohistochemistry (IHC) and in situ hybridization (FISH) were selected. However, thirty patients did not meet the inclusion criteria, including three with a left ventricle ejection fraction (LVEF) < 55% at the beginning of TTZ treatment. In this study, we reported forty-six HER-2-positive breast cancer patients (Figure 1).

### 2.1. Characteristics of the Study Population 

#### Diagnostics

The mean age at diagnosis was 53.6 ± 8.7 years. The physical examination showed significance differences with a weight of 72.7 ± 16 and a body surface of 1.8 ± 0.2 in the CT group instead of a weight of 63.2 ± 9.4 and a body surface of 1.6 ± 0.1 in the group without CT (*p* < 0.05). Seventeen patients had a body mass index > 25 (71.7%; overweight and obese). Concerning the tumor, this was in the left breast in 43.5% of women; at diagnosis, infiltrating ductal carcinoma (IDC) was the most frequent lesion (71.7%) (Table 1). The clinical stages observed in 38 women were II and III; most breast cancer patients were diagnosed in an advanced stage of the disease (82.6%). Regarding hormone receptors (HRs), 22 patients presented positivity (47.8%) (Table 1). 

The anti-HER-2 administration had a triweekly interval (every 21 days). The loading dose showed significant differences, with a median of 540 mg in the CT group versus a median of 440 mg without CT (*p* < 0.05). The maintenance dose was 390 mg, with a duration of 11 months and 17 doses administered (Table 1). 

### 2.2. Risk Factors of Cardiotoxicity in TTZ Patients

The diagnostic criterion used for CT was an absolute drop in LVEF from baseline > 10% or a final LVEF < 53% (ESMO). Baseline LVEF was measured before TTZ administration; the follow-up LVEF was measured 6 ± 1.5 months after TTZ treatment. Cardiotoxicity occurred in 19 patients (41.3%), with a decrease in LVEF of 13%. The patients without CT (27 patients) showed a follow-up LVEF of 62.3 ± 4.2 compared to the CT group, with an LVEF of 56.1 ± 5.5. The cardiotoxicity associated with the decrease in LVEF was statistically significant (*p* < 0.001, Table 1).

Concerning the cardiovascular risk factors (CVRFs), most women with CT had at least five previously reported CVRFs [16] before anti-HER-2 therapy (tobacco consumption, diabetes mellitus II, hypertension, obesity, and hypercholesterolemia), with odds ratios of 13.6, 1.3, 0.9, 2.1, and 1.4, respectively. Smoking was reported in four patients (21.1%); women were active smokers at diagnosis. The patients with a history of hypertension (*n* = 7, 36.8%) and type II diabetes mellitus (*n* = 4, 21.1%) were under pharmacological treatment at diagnosis (Table 1). However, the odds ratio test did not show significant differences in the population. In addition, no association between the tumor location and type of cancer and chemotherapy/taxanes with CT was shown (Table 2). 

## 3. Discussion

Trastuzumab is a humanized monoclonal antibody approved by the Food and Drug Administration (FDA) and used as a standard for the treatment of patients with HER-2-positive BC, as it decreases the risk of relapse (50%) and death (33%), increasing the overall survival of patients by 23–35% [16,17]. However, its use is controversial since previous results showed irreversible adverse effects, ranging from an asymptomatic decrease in the left ventricular ejection fraction to clinically overt heart failure [16,17,18,19].

In the population studied, we observed that cardiotoxicity was higher than expected, with an incidence of 41.3%. Previous studies showed a prevalence of cardiotoxicity from 27% to 30%; the percentage increased up to 34% when TTZ was administered with other chemotherapeutic agents (anthracyclines/taxanes) compared to when prescribed as monotherapy (3–7%) [15,17,18,19]. In Mexico, a recent study showed a prevalence of 46% of cardiotoxicity in a population of Her-positive BC patients treated with a combination of TTZ with anthracyclines. Therefore, in this study, the increased incidence of cardiotoxicity could be associated with the different treatment schemes. In this sense, most breast cancer patients received a concomitant administration of trastuzumab with anthracyclines or taxanes, with cardiotoxic effects previously reported. So, radiotherapy–chemotherapy and TTZ are probably the main factors associated with CT [17,18,19]. The evaluation of cardiac biomarkers is necessary to predict the side effects associated with the combination of TTZ and anthracycline schemes [15,16,20]. Concerning the above, the use of neuro-hormonal blockers (beta-blockers; BBs), angiotensin-converting enzyme (ACE) inhibitors, and angiotensin II receptor antagonists (ARBs) can be implemented as a pharmacological strategy to prevent CT [16,21,22].

The evidence presented in this study suggested that the increased incidence of cardiotoxicity is strongly associated with the combined use of TTZ with different cancer treatments. In this sense, adverse lifestyles can increase oxidative stress and inflammatory processes, playing a central role as risk factors for CT associated with anthracycline use. Smoking, alcohol consumption, sedentary habits, obesity, and type II diabetes mellitus are the main risk factors related to CT [16,17,18,19,23].

However, in the population studied, we found that smoking, hypertension, type II diabetes, and body mass index > 25 (overweight or obesity) were not significant. Indeed, several studies have shown that nutritional status is crucial for the health of breast cancer patients [16,21]. In this sense, a healthy diet has a protective effect concerning cancer prevention and relapses, including cardiovascular disease protection. On the other hand, alcohol intake shows contradictory evidence since moderate consumption is shown as protective, while chronic consumption seems to have a harmful effect. Smoking is the only factor that has an evident detriment in terms of relapsed disease and heart damage (cardiotoxicity) [20]. So, the risk factors reported previously were not associated with CT in this study.

The clinical–pathological characteristics of breast cancer patients showed alarming results. The diagnosis occurred at 54 ± 10 years, which agrees with Maffuz-Azziz et al. [24], who reported an incidence of BC of 54 ± 12 years, and with Gómez et al. [22] in the Uruguayan population, with an age at diagnosis of 55 years. Instead, in Mexico, a recent study reported an age at diagnosis of 50 ± 8 years [15]. However, it differs from other countries with an average diagnosis age of 63. So, we believe that the risk factors presented in the Latin American population are associated with the early age at diagnosis. In addition, we observed a high incidence of infiltrating ductal carcinoma (IDC; 71.7%), a similar percentage to those reported previously [24], showing that IDC is the most frequent cancer (78%). Also, we observed that stage II occurred in 47.8% of patients, followed by III (34.8%), while metastasis (stage IV) was observed only in 2.2% of patients. Cardinale et al. [23] showed that 45.2% of women were diagnosed in advanced stages (II and III), which is an unfavorable prognostic factor. All those results evidence the lack of prognosis and screening tools for BC detection, including the lack of education, awareness, and access to health institutions [22,24].

Hormonal receptors are a determinant in the diagnosis and treatment of BC patients. Our study showed the presence of HR+ in 22 patients (47.8%). However, only 11 records included hormonal therapy in their scheme. These data are consistent with those obtained by Camejo et al. [25], who reported the presence of positive HR in 43% of patients. Those results suggest that a better follow-up of patients with HR+ is necessary since it is an important prognostic factor in the response to treatment and management of the patients [22,24].

The administration regimen is fundamental in cancer treatment. In the population, TTZ was administered three times a week. Previous studies have shown that the scheme used in patients with HER-2-positive BC was 8 mg/Kg with a loading dose and a standard maintenance dose of 6 mg/Kg for one year, with a total of 17–18 cycles, respectively [26,27,28]. These schemes are similar to those used in our study, with an average of 17 doses for 11 months [25,26]. Moreover, the loading dose showed a significant association with CT. In this sense, although there are no previous studies that show a relationship between the loading dose and cardiotoxicity, Earl et al. [27] evaluated the adverse effects following the administration of two TTZ treatment regimens (6 versus 12 months; loading dose of 8 mg/kg; maintenance dose of 6 mg/kg). Authors demonstrated that the 6-month guideline, although it showed the same beneficial results as the 12-month one, assessed for disease-free time in patients (greater than three years in both groups), also caused moderate cardiac events and other toxic events, including cardiotoxicity, but in a lower percentage (3%) than in the 12-month scheme (8%) [27]. Therefore, the authors suggest that the 6-month scheme reduces the adjuvant administration of TTZ. So, we suggested that the loading dose administered could be related to the overweight and obesity observed in the patients, as mentioned previously [21,23]. Limitations of this study include the size of the population and including a more representative population and covering other health centers could show more significant results regarding the characteristics and management of breast cancer survivors; its retrospective design; the combination of different treatment schemes for all patients; and a relatively short follow-up. However, this is the first report on cardiotoxicity after adjuvant and neoadjuvant chemotherapy combined with trastuzumab in southwest Mexican women with HER2-positive breast cancer.

The results obtained in this study confirmed that, in Mexico, it is necessary to perform strategies for Her-2-positive BC patients treated with TTZ to ensure their survival. So, it is essential to carry out an adequate follow-up, including echocardiographic monitoring and the evaluation of cardiac biomarkers, to guarantee safety with the combined treatment of TTZ and anthracyclines. 

## 4. Materials and Methods

### 4.1. Participants and Study Design

This is a retrospective cross-sectional study conducted in an ambulatory care unit (ACU) from 15 January 2015 to 31 July 2019. The ACU mainly focuses on emergency cases of BC in southeast Mexico.

### 4.2. Study Setting

This study was conducted in ACU No. 242 (Tejeria, Veracruz, Mexico), at the Mexican Social Security Institute. Data were collected from the clinical records of all patients diagnosed with HER-2-positive BC. The characteristics of patients were women over 18 years; survivors of HER-2-positive BC, with evidence of QT cycles with TTZ; and clinical records with at least two echocardiograms (one baseline and one follow-up) with the evaluation of LVEF. Patients with a type of cancer other than BC and with a history of heart disease were excluded. Demographic and clinical characteristics of patients, including cardiovascular risk factors, type of BC, and hormonal therapy, were collected.

### 4.3. Echocardiogram and CT Evaluation

In the treatment with TTZ, the echocardiogram must be performed six months after the first anti-HER-2 drug therapy. CT evaluation takes into consideration the results obtained from LVEF. The diagnostic criterion used to define CT was considering a drop in LVEF > 10% from baseline or a final LVEF < 53% [7,14].

### 4.4. Ethical Considerations

This study was approved by the National Commission for Scientific Research, IMSS (registration number R-2019-785-065). All procedures agreed with the ethical standards of the 1975 Declaration of Helsinki and the General Health Law in Mexico. 

### 4.5. Statistical Analysis

A normality variable test was conducted, and data were reported as the mean ± standard deviation or median and interquartile range, according to distribution. The chi-square and Fisher’s exact tests were used to compare categorical variables. The Student’s t-test or Mann–Whitney U test was used for quantitative variables. We performed a logistic regression model for the estimated odds ratio (OR) with their 95% confidence intervals. The significance was set at a *p* value < 0.05. All statistical analyses were performed using the Statistical Package for Social Sciences (SPSS-IBM) version 25.0 (IBM Corp., Armonk, NY, USA).

## 5. Conclusions

Forty-one percent of HER-2-positive BC patients treated with TTZ have CT. Overweight and obesity are dominant predictive factors associated with CT. Although the concomitant use of anthracyclines and TTZ leads to an increase in disease-free life, most patients are diagnosed at advanced stages of cancer. It is crucial to monitor the cardiac function in HER-2-positive BC patients, including a change in their lifestyle, to avoid cardiovascular failure.

## Figures and Tables

**Table 1:** Demographic and clinical characteristics of patients with breast cancer.

	General	Cardiotoxicity		
Characteristics	(*n* = 46)	Yes (*n* = 19)	No (*n* = 27)	*p* Value *
Age (years) [mean ± SD]	53.6 ± 8.7	54.9 ± 10.3	52.33 ± 7.4	0.320
Medical history				
Tobacco consumption, *n* (%)	5 (10.9%)	4 (21.1%)	1 (3.7%)	0.144
Diabetes mellitus II, *n* (%)	6 (13%)	4 (21.1%)	2 (7.4%)	0.213
Hypertension, *n* (%)	15 (32.6%)	7 (36.8%)	8 (29.6%)	0.607
Menopause, *n* (%)	30 (65.2%)	13 (68.4%)	17 (63.0%)	0.783
Hypercholesterolemia, *n* (%)	26 (56.5%)	12 (63.2%)	14 (51.9%)	0.532
Blood cholesterol (mg/dL) [mean ± SD]	209.3 ± 52.1	212.8 ± 56.4	206.7 ± 9.7	0.704
Physical examination				
Weight (kg) [mean ± SD]	67.2 ± 13.4	72.7 ± 16	63.2 ± 9.4	0.028 **
Height [mean ± SD]	153.4 ± 6.2	154.7 ± 5.1	152.4 ± 6.8	0.223
Body surface (m^2^) [mean ± SD]	1.7 ± 0.2	1.8 ± 0.2	1.6 ± 0.1	0.014 **
BMI				
Underweight	1 (2.2%)	0	1 (3.7%)	0.508
Healthy weight	13 (28.2%)	5 (26.3%)	7 (25.9%)	
Overweight	16 (34.8%)	5 (26.3%)	11 (40.8%)	
Obesity	17 (36.9%)	9 (47.4%)	8 (29.6%)	
Cancer description				
Infiltrating ductal carcinoma, *n* (%)	33 (71.7%)	13 (68.4%)	20 (74.1%)	0.675
Clinical stage, *n* (%)				
I	3 (6.5%)	1 (5.3%)	2 (7.4%)	0.461
II	22 (47.8%)	8 (42.1%)	14 (51.8%)	
III	16 (34.8%)	7 (36.8%)	9 (33.3%)	
IV	1 (2.2%)	1 (5.3%)	0	
Not specified	4 (8.7%)	2 (10.5%)	2 (7.4%)	
Tumor side				
Left breast cancer, *n* (%)	20 (43.5%)	7 (36.8%)	13 (48.1%)	0.446
Hormonal receptors				
ER/PRR positive	11 (23.9%)	4 (21.1%)	7 (25.9%)	0.451
ER/PRR negative	24 (52.2%)	10 (52.6%)	14 (51.9%)	
ER−/PRR+	5 10.9%)	1 (5.3%)	4 (14.8%)	
ER+/PRR−	6 (13.0%)	4 (21.1%)	2 (7.4%)	
Oncological treatment				
Mastectomy	46 (100%)	19 (100%)	27 (100%)	1
Radiotherapy	44 (95.7%)	18 (94.7%)	26 (96.3%)	0.798
Radiotherapy + chemotherapy [*n*, %]	41 (89.1%)	16 (84.2%)	25 (92.6%)	0.368
Anthracycline				
No	4 (8.7%)	3 (15.8%)	1 (3.7%)	0.292
Epirubicin + fluorouracil + ciclophosphamide	41 (89.1%)	15 (78.9%)	26 (96.3%)	0.197
Epirubicin + ciclophosphamide	1 (2.2%)	1 (5.3%)	0	
Taxane				
No	9 (19.6%)	4 (21.1%)	5 (18.5%)	0.831
Docetaxel	34 (73.9%)	12 (63.2%)	22 (81.5%)	0.060
Paclitaxel	3 (6.5%)	3 (15.8%)	0	
Both anthracycline and taxane	33 (71.7%)	12 (63.2%)	21 (77.8%)	0.278
TTZ loading dose (mg) [median, IR]	452, 143	540, 200	440, 73	0.041 **
TTZ loading dose (mg/Kg) [median, IR]	7.9, 0.6	7.8, 0.5	7.9, 1.0	0.849
TTZ maintenance doses (mg) [median, IR]	390, 105	432, 140	375, 77	0.100
TTZ duration of treatment (months) [median, IR]	11, 1	11, 3	11, 0	0.137
TTZ doses administered [median, IR]	17, 1	17, 2	17, 1	0.725
				
Heart function				
Basal LVEF [mean ± SD]	63.6 ± 6.4	64.5 ± 8.0	62.9 ± 4.9	0.459
Follow-up LVEF [mean ± SD]	59.7 ± 5.7	56.1 ± 5.5	62.3 ± 4.2	<0.001 *

SD: standard deviation; IR: interquartile range; BMI: body mass index; ER: estrogen receptor; PRR: progesterone receptor; TTZ: trastuzumab; LVEF: left ventricular ejection fraction. * Fisher’s exact test, chi-squared test, Mann–Whitney U test, and Student’s t-test, ** *p* < 0.05.

**Table 2:** Risk factor of cardiotoxicity of TTZ in patients with breast cancer.

Characteristics	CT (*n* = 19)	No CT (*n* = 27)	OR (95% CI)	*p* Value
Age (years)				
<60	15 (78.9%)	24 (88.9%)	1	
>60	4 (21.1%)	3 (11.1%)	4.9 (0.4–64.8)	0.228
Tobacco consumption				
No	15 (78.9%)	26 (96.3%)	1	
Yes	4 (21.1%)	1 (3.7%)	13.6 (0.9–209)	0.061
Diabetes mellitus II				
No	15 (78.9%)	25 (9.26%)	1	
Yes	4 (21.1%)	2 (7.4%)	1.3 (0.08–19.4)	0.865
Hypertension				
No	12 (63.2%)	19 (70.4%)	1	
Yes	7 (36.8%)	8 (29.6%)	0.9 (0.1–5.8)	0.868
Obesity				
No	10 (52.6%)	19 (70.4%)	1	
Yes	9 (47.4%)	8 (29.6%)	1.5 (0.3–6.8)	0.597
Hypercholesterolemia				
No	7 (36.8%)	13 (48.1%)	1	
Yes	12 (63.2%)	14 (51.8%)	2.0 (0.4–10.6)	0.406
Tumor side				
Right breast cancer, *n* (%)	12 (63.2%)	14 (51.9%)	1	
Left breast cancer, *n* (%)	7 (36.8%)	13 (48.1%)	0.6 (0.1–3.6)	0.550
Chemotherapy and taxane				
No	7 (36.8%)	6 (22.2%)	1	
Yes	12 (63.5%)	21 (77.8%)	0.2 (0.1–3.4)	0.276
Taxane				
No	4 (21.1%)	5 (18.5%)	1	
Yes	15 (78.9%)	22 (18.5%)	3.4 (0.2–98.6)	0.478

CT: cardiotoxicity; OR: odds ratio; TTZ: trastuzumab; LVEF: left ventricular ejection fraction.

## Data Availability

The original contributions presented in the study are included in the article, further inquiries can be directed to the corresponding author.
